# Cough induced rib fracture, rupture of the diaphragm and abdominal herniation

**DOI:** 10.1186/1749-7922-1-34

**Published:** 2006-11-24

**Authors:** Andreas Hillenbrand, Doris Henne-Bruns, Peter Wurl

**Affiliations:** 1 Universitiy of Ulm, Department of Viszeral – and Transplantation surgery, Steinhövelstr. 9, 89070 Ulm, Germany

## Abstract

Cough can be associated with many complications. In this article, we present a 59 year old male patient with a very rare combination of a cough related stress fracture of the ninth rib, a traumatic rupture of the diaphragm, and an abdominal wall herniation. The hernia was repaired through surgical treatment without bowel resection, the diaphragm and the internal and oblique abdominal muscle were adapted, and the abdomen was reinforced with a prolene net.

Although each individual injury is well documented in the literature, the combination of rib fracture, abdominal herniation and diaphragm rupture has not been reported.

## Case report

We report a rare case of a cough related stress fracture of the ninth rib, traumatic rupture of the diaphragm and abdominal herniation in a patient with a chronic cough history.

A 59 year old male patient (86 kg; 1,75 m) collapsed at home following intensive coughing. The medical history includes hypertension being treated with a beta-blocker, house-dust-allergy, chronic bronchitis related cough, and two operations on a spinal disc prolaps. The patient is known to have smoked (15 pack years).

There was no previous history of trauma.

**Figure 1:**
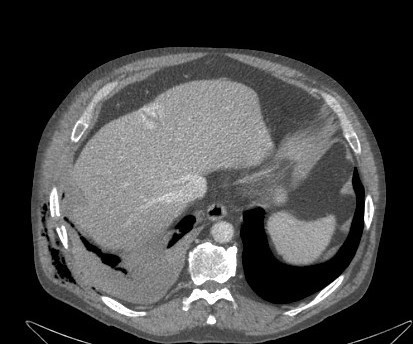
CT scan on admission. Fracture of the ninth right rib with hematothorax and emphysema.

**Figure 2:**
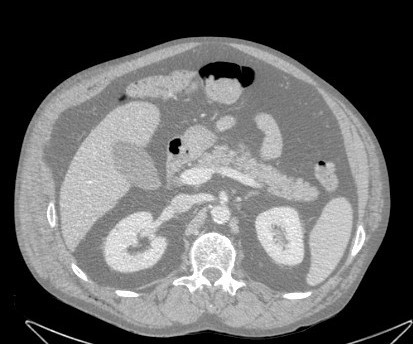
CT scan on admission. No intestinal herniation.

**Figure 3:**
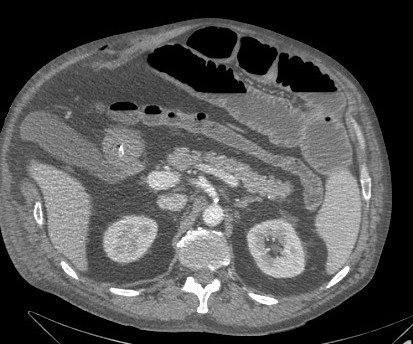
Muscle rupture with intact external abdominal muscle one week after admission.

**Figure 4:**
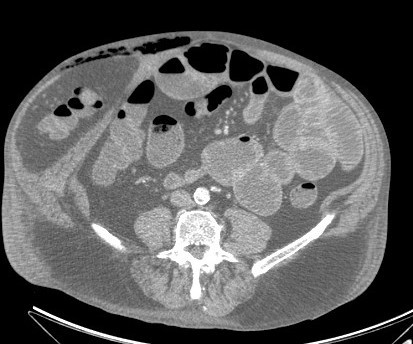
Massive intestinal herniation one week after admission.

On admission to hospital examination revealed a 10 cm well demarcated area of haemorrhage in the right side of the epigastrium. The abdomen was painful, but soft with no palpable mass or herniation. An abdominal computed tomography showed a fracture of the ninth right rib with a surrounding haematoma and hematothorax; however, no bowel herniation or muscle tear was evident (Fig. 1, 2). A thoracic drain was inserted for two days. During the hospital stay the patient's abdomen became meteoristic and painful. He had no bowel movements for five days. A CT scan confirmed an intestinal obstruction, showing an ileus due to a massive herniation on the right lateral side of the abdomen (Fig. 3, 4). An operation followed in which a crosswise incision along the ninth rib was made. The herniation was reduced without bowel resection. During the operation a rupture of the diaphragm also was found. The diaphragm and the internal and oblique abdominal muscle were adapted and the abdomen was reinforced with a prolene net.

Post operation the patient remained intubated for six days to prevent coughing.

At the time of discharge the patient was well. A clinical and radiographic investigation six months later showed no renewed herniation, and the patient remained well.

## Discussion

Violent or sustained coughing can be associated with many complications. The most frequent and best documented complications are rib fractures [1]. Typical locations for rib fractures are the fifth through ninth rib at the lateral aspect of the rib cage. These fractures are caused from opposing muscular forces in the middle of the rib at the axillary line from the serratus anterior and external oblique muscles [2]. Other cough induced rib fractures are caused by a complex interplay between inspiratory and exspiratory muscles. Serious complications are rare and may involve pneumothorax [3], bleeding [4] or even intercostal pulmonary hernia [5]. Therapy for sole rib fracture is conservative with treatment of the cough causing factor.

The diaphragm is mainly an inspiratory muscle, but it also contracts during the expiratory phase of a cough [6]. During forced respiratory movements, the muscles of the abdominal wall contract pushing the diaphragm upward whereas the ribs are pushed inward and downward. This kind of opposing action can result in diaphragmatic rupture with a consequent herniation of bowel loops into the chest.

Defects of the abdominal wall after coughing are rare and require a surgical intervention [7]. Both abdominal herniations as well as abdominal muscle tears were reported. Abdominal muscle tears are frequently misdiagnosed due to their mimicry of an acute abdomen, appendicitis or all kinds of gynaecological diseases and emergencies [8]. A computed tomography seems to be essential for an accurate diagnosis [9]. Abdominal muscle tears are generally most common in middle-aged and elderly patients with chronic bronchitis [10]. In contrast to the abdominal muscle tears, abdominal herniations caused by cough are in general easier to detect, but they commonly appear delayed [11].

In summary, since both the diaphragm and abdominal muscles are attached to the lower ribs, opposing forces can result in a rib fracture, diaphragmatic rupture and abdominal herniation due to cough.

Coughing can be associated with many complications. Rib fractures are easily diagnosed, but abdominal muscle tears are frequently missed. They usually appear delayed and a computed tomography seems to be essential for an accurate diagnosis.

Although each individual injury is well documented in the literature, the combination of rib fracture, abdominal herniation and diaphragm rupture however has not been reported so far.
